# Machine learning accurately classifies age of toddlers based on eye tracking

**DOI:** 10.1038/s41598-019-42764-z

**Published:** 2019-04-18

**Authors:** Kirsten A. Dalrymple, Ming Jiang, Qi Zhao, Jed T. Elison

**Affiliations:** 1 Institute of Child Development, University of Minnesota, Minneapolis, USA; 2 Computer Science and Engineering, University of Minnesota, Minneapolis, USA; 3 Department of Pediatrics, University of Minnesota, Minneapolis, USA

**Keywords:** Machine learning, Human behaviour

## Abstract

How people extract visual information from complex scenes provides important information about cognitive processes. Eye tracking studies that have used naturalistic, rather than highly controlled experimental stimuli, reveal that variability in looking behavior is determined by bottom-up image properties such as intensity, color, and orientation, top-down factors such as task instructions and semantic information, and individual differences in genetics, cognitive function and social functioning. These differences are often revealed using areas of interest that are chosen by the experimenter or other human observers. In contrast, we adopted a data-driven approach by using machine learning (Support Vector Machine (SVM) and Deep Learning (DL)) to elucidate factors that contribute to age-related variability in gaze patterns. These models classified the infants by age with a high degree of accuracy, and identified meaningful features distinguishing the age groups. Our results demonstrate that machine learning is an effective tool for understanding how looking patterns vary according to age, providing insight into how toddlers allocate attention and how that changes with development. This sensitivity for detecting differences in exploratory gaze behavior in toddlers highlights the utility of machine learning for characterizing a variety of developmental capacities.

## Introduction

There are several sources of inter-individual variability in human gaze patterns, such as genetics^1,2^, social functioning^3,4^, cognitive functioning^5^ and age^6,7^. These differences are often revealed using areas of interest (AOIs) that are chosen by the experimenter or other human observers. We adopted a data-driven approach for characterizing gaze patterns by using a combination of SVM and DL techniques to classify 18- and 30-month-old infants (final sample n = 41) by age based on their exploratory gaze patterns, which were recorded while they viewed static images of natural scenes. We chose these age groups because (1) social and symbolic communication changes dramatically over this period and, (2) delays in social and symbolic communication during this developmental period can represent risk factors for autism spectrum disorder^8^. Differences in fixation patterns could reflect meaningful changes in how toddlers process information about the world around them.

**Figure 1:**
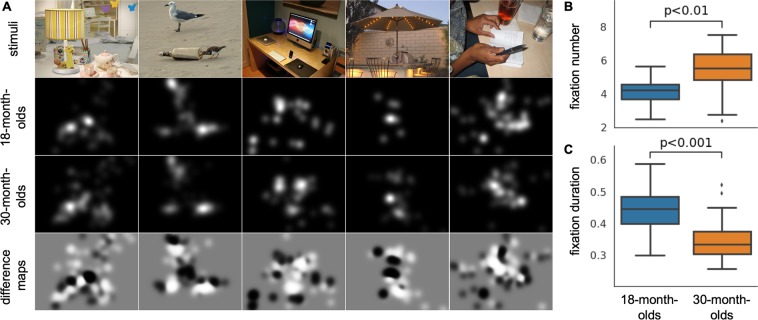
Basic eye movement metrics. (**A**) Examples of the visual stimuli, fixation maps of each age group, and difference maps. In the difference maps, bright regions attract more fixations from the 30-month-olds, while dark regions attract more fixations from the 18-month-olds. (**B,C**) Significant differences between age groups were observed. (**B**) 30-month-olds had more fixations than 18-month-olds. (**C**) 30-month-olds had shorter average fixation durations than 18-month-olds.

Images consisted of social and non-social scenes that included multiple dominant objects, rather than a central dominant object^9^ (Fig. 1A). For example, rather than depicting a social object, such as a person, front and center, an image might consist of non-human objects in the foreground with a person in the background. Thus, semantically salient objects (such as faces) are not always the most visually salient (low-level) features in the scenes, removing a common confound in scene viewing research. Multiple dominant objects in the same scene also create the opportunity for a statistical analysis on the relative importance of objects or features in gaze allocation. These images provide visual information more similar to real-world free viewing than traditional, controlled experimental stimuli that often display only the objects of immediate interest, and allow more equitable competition between low-level image salience (e.g. pixel-level salience) and high level, semantic salience.

Toddlers (n = 73) viewed 100 naturalistic images for 3 seconds each while their eye movements were tracked using a Tobii TX300 eye tracker (Tobii Technology AB, www.tobii.com). We first assessed data quality by generating measures of calibration accuracy and precision using the procedure described in a previous study^10^. Using only data from recordings with acceptable calibration accuracy (18-month-olds n = 19; 30-month-olds n = 22, see Methods), we looked at the basic eye movement metrics of fixation number and duration. A two-tailed unpaired t test showed that 18-month-olds made fewer fixations than 30-month-olds (18-month-olds: mean = 5.96, SD = 0.84; 30-month-olds: mean = 6.81, SD = 0.87, Fig. 1B, t(39) = −3.151, p = 0.003, 95% CI [−1.4–0.3], Cohen’s d = −0.987). We also observed that 18-month-olds had longer fixation durations than 30-month-olds (18-month-olds: mean = 433.93 ms, SD = 74.45; 30-month-olds: mean = 348.63 ms, SD = 68.71, Fig. 1C, t(39) = 3.814, p < 0.001, 95% CI [40.1 130.5], Cohen’s d = 1.194). We used the fixation information to generate fixation maps of the two groups (Fig. 1A). These fixation maps indicate where toddlers from each age group fixated and allowed us to compute difference maps highlighting the key areas within the naturalistic scenes that distinguish the looking patterns of the groups. The primary differences are that 18-month-olds show greater interest in faces and the 30-month-olds show greater interest in things that are gazed at, or touched, by others. This difference between age groups could reflect changes in cognitive representation of others’ intentionality.

**Figure 2:**
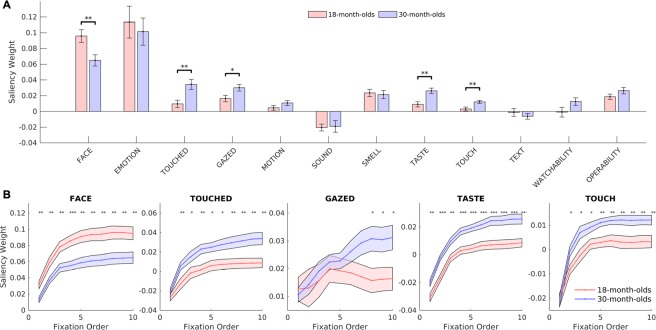
Saliency weights of semantic features. (**A**) Saliency weights of semantic features. The 30-month-olds had a greater interest in objects being touched or gazed. Error bars denote the SEM over the group of toddlers. Asterisks indicate significant difference between 18-month-olds and 30-month-olds using a two-tailed unpaired t test. *p < 0.05, **p < 0.01. (**B**) Evolution of saliency weights of the five semantic features with the most significant differences between age groups. Fixations start with first fixation away from the center fixation dot post-stimulus-onset. Shaded areas denote ± SEM over the group of toddlers. Asterisks indicate significant difference between people with ASD and controls using a two-tailed unpaired t test. *p < 0.05, **p < 0.01, and ***p < 0.001.

Beyond analysing basic fixation metrics and looking patterns, our research method consists of two different computational approaches: (1) a group-wise analysis based on predefined features of interests, and (2) a DL-based classifier for estimating the age group of individual toddlers. First, we conducted computational and statistical analyses to identify the group-wise differences between 18-month-olds and 30-month-olds. To conduct a better-controlled statistical analysis, for each toddler, we trained a linear SVM model to classify salient vs. non-salient image regions using a set of predefined features of interest. The combination weights of the various features indicated their respective contributions to attention allocation (namely saliency weights), which were compared between the two age groups. The saliency model used five categories of predefined feature maps: (1) pixel-level features (e.g. color, intensity, orientation), (2) object-level features (e.g. size, complexity, etc.), (3) semantic-level features (e.g. faces, emotional expressions, objects touched by a human or animal, etc.), (4) image center, and (5) background^9^. In our previous studies, these features have showed their reliability in predicting eye movements^9^ and identifying people with autism^4^. We trained the saliency model using gaze patterns from each child and then compared feature weights between 18-month-olds and 30-month-olds to determine if differences exist between the groups. The comparison indicated that the two groups differed from each other in meaningful ways: while both the 18- and 30-month-olds looked at faces early on, the 18-month-olds looked at faces more, and continued to fixate faces across subsequent fixations (18-month-olds: mean = 0.096, SD = 0.035; 30-month-olds: mean = 0.065, SD = 0.033, t(39) = 2.881, Fig. 2A, p = 0.006, 95% CI [0.009 0.053], Cohen’s d = 0.902). In contrast, the 30-month-olds ultimately explored more of the scene, showing interest in the objects of other people’s gaze (18-month-olds: mean = 0.016, SD = 0.018; 30-month-olds: mean = 0.030, SD = 0.019, Fig. 2A, t(39) = −2.365, p = 0.023, 95% CI [−0.026–0.002], Cohen’s d = −0.741), objects being touched by a human or animal (18-month-olds: mean = 0.010, SD = 0.020; 30-month-olds: mean = 0.034, SD = 0.030, Fig. 2A, t(39) = −3.042, p = 0.004, 95% CI [−0.041–0.008], Cohen’s d = −0.953), objects that elicit strong tactile sensations (e.g. sharp knife, soft pillow; 18-month-olds: mean = 0.003, SD = 0.011; 30-month-olds: mean = 0.012, SD = 0.009, Fig. 2A, t(39) = −2.896, p = 0.006, 95% CI [−0.015–0.003], Cohen’s d = −0.907), and objects that can be tasted (e.g. food and drink; 18-month-olds: mean = 0.009, SD = 0.014; 30-month-olds: mean = 0.026, SD = 0.017, Fig. 2A, t(39) = −3.519, p = 0.001, 95% CI [−0.027–0.007], Cohen’s d = −1.102). These differences between groups emerge early, often within the first 1–3 fixations (excluding the first fixation after stimulus onset) (Fig. 2B) and represent semantic categories that reflect meaningful development of cognitive processing (see Discussion). Figure 2B illustrates how differences emerged in the groups across fixation number and highlights the importance of analyzing eye movement data across time, rather than simply extracting an outcome based on aggregate data from entire trials.

**Figure 3:**
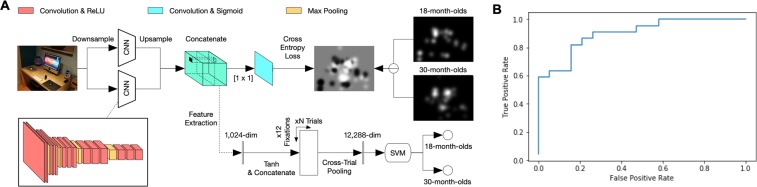
Deep Learning model. (**A**) An overview of the DL model, which was composed of two parallel convolutional neural networks (CNNs) encoding two scales of visual input to extract high-level representations of an image and predict the corresponding difference map between fixations of 18-month-olds and 30-month-olds. At each fixation point, a 1024-dimensional feature vector was extracted from the convolutional layers. The feature vectors were integrated across trials to represent each toddler’s eye-tracking patterns. These representations were classified with a linear SVM to distinguish the two age groups. (**B**) The ROC curve of the DL classification. Positive values indicate 30-month-olds; negative values indicate 18-month-olds.

**Figure 4:**
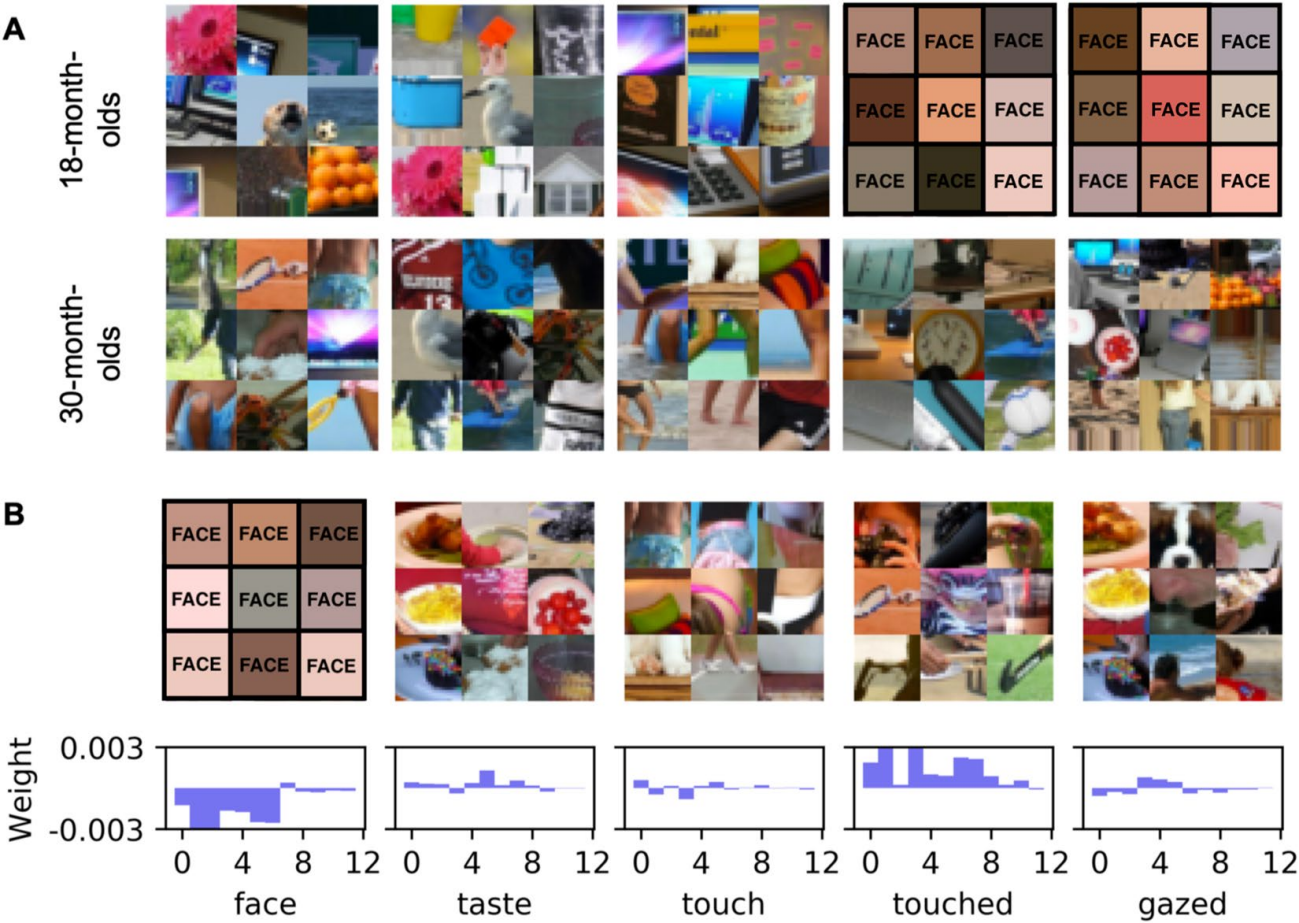
Visualization of DL features. (**A**) Visualization of the DL feature channels sorted into columns corresponding to classification weights. The lowest weights (row 1) correspond to 18-month-olds and the highest weights (row 2) correspond to 30-month-olds. Image patches represent features that are most characteristic of looking patterns for each age group across all fixations. (**B**) Visualization and classification weights of the DL features corresponding to each feature from Fig. 2B. Features that most strongly correlate with the labeled semantic features are presented. Bars indicate the evolution of classification weights across fixations. Higher classification weights suggest that 30-month-olds are more likely to fixate the feature than 18-month-olds. Note: for privacy reasons, faces are blocked in this Figure. Faces were intact in the original stimuli viewed by participants.

In the aforementioned weight analysis, we used predefined features at multiple levels and analyzed their relative importance in eliciting gaze in the two age groups of interest. Given that differences exist between the two groups of gaze patterns, our next exploration uses Deep Learning (DL) to automatically learn and visualize the hierarchical features from the images and eye-tracking data in a data-driven manner without any hypothesis or human design of features. In particular, by fitting the difference between the fixations of 18-month-olds and 30-month-olds, our DL model (Fig. 3A) learns multi-level image features that distinguish the two age groups. A difference map was first computed with a subtraction between the fixation maps of the two age groups. The model was composed of two parallel convolutional neural networks (CNNs) encoding two scales of input that extract multi-level image properties and then predict the corresponding difference map. At each fixation point, a 1024-dimensional feature vector was extracted from the convolutional layers. These features were aggregated across all experimental trials of each toddler. Figure 4A provides visualizations of the features that best represent the gaze patterns of 18- and 30-month-olds. Like the saliency weight analysis, DL revealed that 18-months-olds spent more time gazing at faces, while 30-month-olds fixated more diverse image features, such as the objects of people’s gaze (e.g. text). It is also interesting to note that the predefined feature maps were categorized by adult raters (see 9) yet did a reasonably good job of classifying toddlers gaze position (Fig. 4B).

Finally, we used a linear SVM classifier on the features extracted by our DL model for the classification of age groups. The goal was to find a linear decision boundary with a maximum margin separating the two groups of toddlers. The ROC curve of our DL classification indicates a high degree of classification accuracy (0.83, AUC = 0.90, sensitivity = 0.81, specificity = 0.84, Fig. 3B). For comparison, our baseline model using simple gaze-based linear SVM without DL had an accuracy of 0.68 and AUC = 0.86 (sensitivity = 0.68, specificity = 0.68). Taken together, our model with DL for feature learning and SVM for classification produces highly accurate classifications. It also reveals features that drive that classification, consistent with conclusions from the saliency weight analysis. These features represent areas within the images that attract the toddlers’ attention differently depending on the age of the toddler and could therefore provide information about cognitive changes that are taking place during this period of development.

The current study demonstrates the efficacy of using SVM and DL for classifying toddlers based on their fixation patterns during free viewing of naturalistic images. The models identified meaningful image features that differentiated the two groups, such as faces, objects of others’ gaze, objects that can be touched, objects that elicit strong tactile sensations, and things that can be tasted. The combination of eye-tracking, DL, and SVM to investigate age-related variability in toddlers represents a new application of the methodology.

The SVM and DL techniques are unique in that they take looking patterns from individuals scanning very complex images and analyze them according to both pixel-level (bottom-up) salience, and semantic-level (top-down) salience. In other words, while some models focus on *either* bottom-up^11^ *or* top-down information^12,13^ for analyzing looking patterns across natural scenes, these techniques use low-level *and* high-level information from the scenes, in addition to where individuals look, to accurately classify observers according to age. This combination of selection mechanisms represents a more accurate account of how attention is actually deployed during visual exploration^14^. We used a probability map to identify group-level differences between 18- and 30-month-olds, from which the DL model can learn discriminative image features that maximally distinguish the two age groups. Compared with training a separate network for each group and combining their features for classification (AUC = 0.85, accuracy = 0.83, sensitivity = 0.86, specificity = 0.79), our DL model trained on the difference maps performed better with less computational cost.

Although DL has been criticized in the past for being a “black-box” in that it lacks transparency regarding how it generates its classifications^15,16^ we are applying visualization techniques to determine which critical image features distinguish the looking patterns of the two groups. In other words, rather than simply determining that age-related variability in toddlers can be captured using DL by analyzing their gaze patterns, this technique is providing meaningful insights into what distinguishes the groups, particularly *which image features* tend to be fixated differently by the two groups. The data-driven DL model converged on similar features to the saliency weight model, which used a three-layer saliency model including semantic-level features identified by human raters^9^. Although DL has been shown to effectively predict gaze behavior in humans before^17–22^ this study goes a step further by using SVM and DL *together* to classify toddlers according to age, while additionally revealing meaningful information about how the two groups allocate their attention differently to features within the scenes.

Researchers have investigated gaze patterns in toddlers, and it is clear that the complexity of the images they are shown matters. Bornstein, Mash, and Arterberry^23^ recorded looking patterns of 4-month-old infants as they examined natural scenes and compared those patterns to viewing patterns directed towards artificial (experimental) scenes. They discovered that infants treat objects and context differently in naturalistic scenes compared to experimental scenes. Pushing ecological validity a step further, others have shown infants dynamic scenes (videos) and reported differences between scanning patterns based on age^6,7^. However, features in these studies were coded according to broad experimenter-determined categories (AOIs) such as faces, hands, and salient objects, and groups were compared in terms of percentages of gaze directed to each category. Although our stimuli are static, they consisted of social and non-social scenes that included multiple dominant objects, rather than a central dominant object, in the same scene, providing visual information more similar to real-world free viewing than traditional, controlled stimuli. By using naturalistic images we are getting closer to determining how toddlers distribute gaze in the real world.

We used static images in this work as they are more controlled than dynamic stimuli. Importantly, image saliency models using static images have been much more explored in the literature, providing better platforms for the study, and allowing more delicate and comprehensive comparisons of features of interest. Our approach could be applied to dynamic stimuli with straightforward extensions (i.e., learning spatio-temporal features instead of spatial features, and increased computational cost). Recent deep neural networks have successfully extended gaze prediction from images to videos^24–27^. These models are able to encode complex and dynamic visual cues, making it feasible to extend our approach for video stimuli in future studies.

The features that are fixated differently between 18- and 30-month-olds in this study map onto meaningful developmental changes during this age period. In particular, language production and comprehension changes dramatically during this period^28,29^, perhaps augmenting the explicit representation of others’ intentionality. This change is reflected in the transition from looking primarily at faces at 18-months, to looking at the objects of other people’s gaze, and items that are touched by others, at 30-months. In contrast to the 18-month-olds, the 30-month-old toddlers appear to be interested not only in the people in the scenes, but also what these people are attending to, and actions that could be implemented. These findings are consistent with results from other studies using natural scenes with toddlers^7^. Using predefined areas of interest, Frank, Vul, and Saxe^7^ found a systematic change in what children ages 3- to 30-months looked at in dynamic stimuli. Infants looked at stimuli differently depending on whether they consisted of a single face, a whole person, or complex scene, but in particular, older children looked at hands in the complex scenes more than the younger children.

One consideration is whether the differences between 18- and 30-month-olds reported in the present study could be driven by differences in information processing speed between the two age groups. Although we did not include a procedure that would allow us to characterize information processing speed, Fig. 2B suggests that information processing speed alone cannot explain the differences in gaze patterns. If information processing speed were driving the differences between groups, we would expect that the 18-month-olds would show a lagged pattern of similar features to 30-month-olds (i.e. the 18-month-olds would fixate the same things as the 30-month-olds, but on later fixations).

In summary, leveraging a model-based eye-tracking approach, in combination with machine learning techniques, we elucidated age related variability in gaze patterns to natural scenes during an important developmental period. This work illustrates an analytic approach that emphasizes prediction^30^. However, the approach does not neglect explanation, as DL revealed specific features of a complex scene that differentiate gaze patterns between 18- and 30-month-olds. The capability to accurately predict age based on gaze patterns over a relatively short window of time has broad implications for the characterization of age-related variability in other psychological domains as well as developmental delays in various aspects of social cognition.

## Methods

### Participants

We recruited thirty-seven 18-month-old (12 male, mean age = 18.59 months, range 18.1–18.97) and thirty-six 30-month-old (19 male, mean age = 30.42 months, range 30.0–30.9) toddlers by email or by phone through the research participant registry at the Institute of Child Development at the University of Minnesota. Upon arrival, the experimenter explained the study in detail, and parents signed informed consent forms to confirm their willingness to have their child volunteer in the study. Parents were compensated for their time with a gift card. This study was approved by the Institutional Review Board at the University of Minnesota. All experiments were performed in accordance with the ethical standards declared in the 1964 Declaration of Helsinki and its later amendments.

### Stimuli

We selected one hundred images from the 700 images used in^4^. These images were acquired from the Object and Semantic Images Eye-tracking (OSIE) database^9^ and selected based on their discriminative ability in Wang *et al*.^4^ and their level of developmental suitability for toddlers as judged by members of the research team.

### Procedure

Toddlers were seated in their parent’s lap approximately 65 cm from a 1920 × 1080 resolution ASUS monitor that subtended 43.6 degrees of visual angle. Eye gaze was recorded using a Tobii TX300 eye tracker (Tobii Technology AB, www.tobii.com). Parents were instructed to sit quietly with their child on their lap. After a 5-location calibration, we implemented a calibration verification procedure, which involved placing 5 additional targets sequentially on the screen (see *Data Quality Assurance* below, and 14). The experimenter then explained to the parent that images were going to appear on the screen and that they should not point to or name anything on the screen. Toddlers were instructed to look at the pictures. Background music played while 1440 × 1080 pixel images were presented on a black screen in random order. Images were divided into two blocks of 50 images, with a short (1–2 minute) break between blocks. Images were displayed for 3 s each. Between images, a grey screen with a white fixation shape appeared to attract the child’s attention to the center of the display. When the experimenter judged that the child was looking at the fixation shape, she pressed a key to initiate the presentation of the next image. The experimenter varied the timing of the key press to discourage toddlers from making anticipatory eye movements before the next image appeared on the screen. Dynamic audio-visual stimuli were presented in the center of the screen every 7–8 trials to help maintain the child’s attention. Each block took approximately 4 minutes and the task took approximately 10–15 minutes from start to finish, depending on how long it took to achieve a good calibration, whether the child was attentive or fussy, and whether the child needed a break between blocks.

### Data Quality Assurance

We performed a calibration verification procedure^10^ before implementing our scene viewing task. This verification procedure involved placing colorful dots on the screen at known locations so that we could later compute calibration accuracy and precision for each toddler. This allowed us to remove the data from any toddler with unacceptably large calibration error. Using data collected during the calibration verification procedure, we first calculated the distance between the recorded point of gaze for each toddler from the center of each target as a measure of calibration accuracy according to the method described in Dalrymple *et al*.^10^. Of the 73 toddlers that we tested, data from fifteen 18-month-olds and fourteen 30-month-olds were rejected because these individuals had fewer than 3 of 5 valid trials during our calibration verification procedure, making it difficult to confidently measure the accuracy of their calibration. Using data from the remaining 44 toddlers, we computed mean accuracy scores in degrees of visual angle for each verification target. We then removed any trials where calibration accuracy was greater than 2 standard deviations above this mean accuracy (greater = more error). Finally, we removed data from three 18-month-olds participants who had fewer than 3 remaining valid verification trials out of 5.

Although this procedure removed a large number of toddlers, this conservative approach ensured that there were no significant differences in calibration accuracy between 18- and 30-month-old infants: the remaining 18-month-olds (n = 19) had a mean calibration error of 2.09°, with RMS precision of (0.23, 0.24), which did not differ from the mean accuracy (1.73°), or precision (0.21, 0.25) from the remaining 30-month-olds (n = 22), *p* > 0.45. This is critical because differences in calibration accuracy between groups could create misleading differences between group looking patterns. Supporting this assertion, the model’s classification accuracy was indeed higher when analyzing the full sample (n = 73, Accuracy = 0.88, AUC = 0.92) compared to the reduced sample (n = 41, Accuracy = 0.83, AUC = 0.90).

### Gaze Data Processing and Fixation Detection

Binocular gaze data were resampled at 1k Hz and the average gaze position of the two eyes was used for fixation detection. Fixations were characterized with a non-parametric algorithm (Cluster Fix^31^) based on K-means cluster analysis. Trials where the algorithm failed to detect any fixation within the image region were excluded. To differentiate salient and non-salient elements in the images, we computed fixation maps for each toddler, as well as the aggregated maps for each group, by plotting a Gaussian blob at each fixation position. The fixation maps indicate the probability distribution of eye fixations in each image.

### Computational Model for Visual Saliency Analysis

We analyzed the eye fixations using a linear SVM model for saliency prediction. The goal in this analysis was to learn the SVM weights of various features that determine the contribution of respective features in driving gaze by classifying salient and non-salient elements in each image. The SVM model incorporates five categories of feature maps: pixel-, object-, and semantic-level features, image center, and background. The use of these predefined features is inspired by the findings that human visual attention is consistently attracted by (1) low-level features such as contrasts^32^, edge content^33^, intensity bispectra^34^, and color^35^; (2) object-level features^36–40^ and (3) semantic-level features, such as faces^41–45^, emotion^46^, gaze directions^47,48^, tactile directions^49^, implied motion^50–52^, other perceptions (such as sound, smell, taste, and touch)^53,54^, text^45^ and tools^55,56^. Specifically, we computed pixel-level feature maps (i.e., color, intensity, orientation) using the well-known Itti-Koch saliency model^57^. Objects in the images were manually segmented and five object-level features were computed for each object: size, convexity, solidity, complexity, and eccentricity. These objects were also annotated with four categories of 12 semantic attributes: (1) directly relating to humans (i.e., face, emotion, touched, gazed), (2) objects with implied motion in the image, (3) relating to other (non-visual) senses of humans (i.e., sound, smell, taste, touch), and (4) designed to attract attention or for interaction with humans (i.e., text, watchability, operability). We generated object- and semantic-level feature maps by placing a 2D Gaussian blob (σ = 2°) at each object’s center. The magnitude of the Gaussian was either the calculated object-level feature value, or manually labeled semantic-level feature value. In addition, the image center map was defined as a single Gaussian blob (σ = 4°) at the center position, representing the center bias. The background map was defined as a binary mask of the unlabeled image regions. For more details of these features, please refer to our previous studies^8,9^.

An SVM classifier was trained to linearly combine the predefined feature maps to fit each toddler’ fixation maps. Thus, the parameters of the learned classifiers represented the relative contribution of each feature in predicting gaze allocation, namely the saliency weights. To train this model on the ground-truth human fixation maps (plotting all fixation points with a Gaussian blur, σ = 1°), 100 pixels in each image were randomly sampled from the 10% most fixated regions as positive samples, and 300 pixels were sampled from the 30% least fixated regions as negative samples. All samples were normalized to have zero mean and unit variance in the feature space. A schematic flow chart of the model is detailed in Fig. 2A. Importantly, separate classifiers were trained individually (and hence saliency weights derived individually) for each toddler (and each fixation in the time course analysis), permitting statistical comparisons between 18- and 30-month-olds. After training, the saliency weights were normalized so that for each toddler they summed to one. The saliency weights were compared between the two age groups using a two-tailed unpaired t test and a Bonferroni correction^58^.

### Deep Learning for Classification of Age Groups

The DL-based approach classifies individual gaze patterns in the following three steps: First, we trained a deep neural network model on the difference of fixation maps between 18-month-olds and 30-month-olds. We computed the between-group difference maps by subtracting one fixation map from the other, as demonstrated in Fig. 1A, where bright regions in the difference maps indicate more fixations of the 30-month-olds, while darker regions indicate more fixations of the 18-month-olds. The network architecture followed the design of the SALICON network^17^. It takes the full-resolution images as input, and computes a hierarchy of features using two parallel VGG-16 backbones^59^ that encode two scales (i.e., one full-resolution and the other down sampled by half). Each VGG-16 network has 13 convolutional layers and the final convolutional layer outputs 512 feature maps. The two sets of feature maps were rescaled to the same resolution (38 × 50) and linearly combined with 1024 parameters. The combination results were transformed with a sigmoid function into a probability map to predict the eye fixation differences. The model parameters were initialized to the pre-trained parameters on the ImageNet^60^ and then fine-tuned on the 100 images with a stochastic gradient descent optimizer. A pixel-wise cross entropy loss function was used as the optimization criterion, which measures the difference between the prediction and the true fixation difference maps.

Next, we represented each toddler with features extracted from the fine-tuned neural network. The neural network generated 1024 feature maps to distinguish the two age groups. At each fixation point, a 1024-dimensional vector was extracted from the feature maps and a tanh transformation was applied. These features were aggregated across all eye-tracking trials to encode what each toddler fixated. Specifically, within each trial, features at all fixation points were concatenated first, and then the concatenated features of all trials were averaged along each feature dimension.

Finally, a linear SVM classifier was trained on the extracted features, to find a linear decision boundary with a maximum margin separating the two groups of toddlers. We used an L2 regularization with the penalty parameter C = 1 to train the SVM classifier. During the testing phase, the trained SVM made a classification for the age group of each toddler, while providing a corresponding probability that allowed us to introduce a flexible threshold to determine the final classification labels. The classification performance was assessed with a leave-one-subject-out cross-validation (i.e., in each run, we used one toddler as the test sample, and all the other toddlers as the training samples). We computed the classification accuracy, specificity (i.e., true positive rate), and sensitivity (i.e., true negative rate), and the area under the ROC curve (AUC) as quantitative evaluation measures.

### Single-Trial Classification

**Figure 5:**
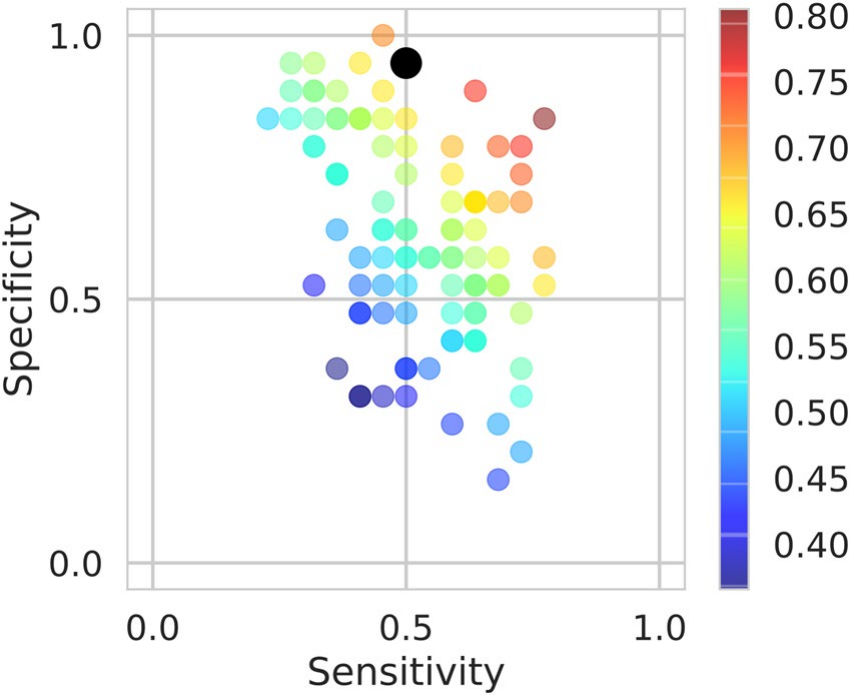
Single-trial classification results. Distribution of the single-trial classification performances. Each point indicates the sensitivity (0.52 ± 0.14, mean ± SD) and specificity (0.62 ± 0.19, mean ± SD) of a single-trial classifier, while its color indicates the classification accuracy (0.57 ± 0.09, mean ± SD). The black point indicates the majority voting result (accuracy = 0.70).

We further analyzed and compared the performances of classifiers trained on each single image. For each toddler, neural network features at all fixations in the same image were extracted and concatenated. Instead of averaging them across trials, in the single-trial classification, a linear SVM classifier was trained to classify eye fixations in each of the 100 trials. Similar to above, all classifiers were trained with an L2 regularization with the penalty parameter C = 1, and a leave-one-subject-out cross validation was performed. The single-trial classification results were combined using a majority voting rule. Figure 5 illustrates the distribution of the single-trial classification accuracies and images with the top and bottom classification accuracies.

### Visualization of DL Features

Network interpretation builds an interpretable prototype (e.g. the original image patches that are selected to best represent the features) in the input domain that represents the abstract concept learned by a neural network (see a recent review by Montavon *et al*.^61^). A typical interpretation method is activation maximization – searching for an input pattern that produces a maximum neuronal response. A direct way to search for the optimal input pattern is to seek it from the input data, while more sophisticated methods generate novel image patterns from optimal neural “codes” using backpropagation or generative models. In this work, we visualized the learned DL features and searched for an interpretation of these features by analyzing their weights in the SVM classifier. Features with positive and negative weights supported the classification of 30-month-olds and 18-month-olds, respectively. We selected the features with the highest absolute classification weights, and presented 128 × 128 image patches at nine image positions that best activate the neurons in the corresponding feature channels. In Fig. 4, we showed these image patches as qualitative examples of which features were most characteristic of the looking patterns of each age group. Because the SVM classifier linearly combines the neuron activations, the stronger the activation and the higher the absolute classification weight, the more important the corresponding image region will be to the classification. Thus, with the highest activations and classification weights, the selected image patches are quantitatively optimal. Compared with the other methods, our method provides direct mappings from input image regions to classification outputs, making it easier to understand how different input regions contribute to the classification.

## Data Availability

Requests for further information should be directed to, and will be fulfilled by, Lead Contacts Kirsten Dalrymple (kad@umn.edu) or Jed Elison (jtelison@umn.edu). Resources and dataset can be retrieved at https://osf.io/v5w8j.
